# Supplementary material for: Labelling affects agreement with political statements of right-wing populist parties
Source: PLoS One. 2020 Nov 11;15(11):e0239772. doi: 10.1371/journal.pone.0239772 (PMC7657482; doi:10.1371/journal.pone.0239772)
Supplement: Attachment — Submitted filename: Response to Reviewers.docx [file pone.0239772.s001.docx]

**Dear Dr. Ha,**

**We submit here our revised manuscript titled “Labelling affects agreement with political statements of right-wing populist parties” (PONE-D-20-15712).**

**We appreciate the assistance and the constructive suggestions the reviewers provided. We have revised the manuscript accordingly and copied and pasted the revised or added text into this response letter. In the main manuscript, any revised or newly inserted text is featured in red highlight.**

**Thank you for your time and assistance with this process.**

**Reviewer 1**

This is an interesting and well-written short manuscript, one that reports the findings of a study examining whether ‘mere labelling political messages’ changes the way in which recipients receive the message in question. More specifically, the study being reported examined whether people would respond differently (agree/disagree) to populist messages depending on whether or not the message was framed as originating from a populist party. The findings reported in this paper are compelling and confirm that this was the case. And as far as I can judge this (as someone with modest expertise in regression analyses) the methods being deployed seem fine.

I very much like the paper’s main argument, and welcome this contribution because it fits nicely within a growing body of research in social and organizational psychology. That being said, there is also clear room for improvement, and in the remainder of this review I will list three issues that, in my view, would need to be addressed before this manuscript could be deemed suitable for publication in PLOS-One.

**We thank the reviewer for this encouraging and positive feedback and appreciate the detailed and constructive comments provided. We have addressed the reviewer’s suggestions as outlined in the following.**

1. The authors claim ‘to be the first ones to examine the effect of labelling on political messages’. If we take this statement literal (‘labelling’) then it could be argued that this statement is correct. However, there are many other studies that have shown that the persuasiveness of a message depends on the (perceived) source, and when we take that into account, the statement is incorrect. This problem is easily fixed, and would merely involve refraining from making this, in my view inaccurate, statement.

**We agree with the reviewer and have therefore deleted the statement.**

**In addition, we now state that we examine labelling in the context of source information. On page 3, we write, “We thus examine whether agreement with the very same statement changes when a right-wing populist party label is affixed to it (i.e., varying the source information) and whether individuals’ political orientation moderates corresponding labelling effects on agreement.”**

2. Following on from the first point, the paper is under-referenced, and misses several highly relevant contributions already showing that a message will be received very differently depending on what source the message is believed to come from. For example, this idea is discussed in quite some detail in Jonathan Haidt’s 2012 book ‘The Righteous Mind’, in which Haidt recalls David Hume’s insight that “emotion and empathy precede reason”, (i.e. translated into plain English: we first lean one way or the other emotionally, and we subsequent retrofit reasons onto this emotion.

**This is useful advice—and we agree that it is inspiring and fruitful to relate our research to Jonathan Haidt’s basic ideas about intuitive judgments. Nonetheless, we refrained from referring to Haidt’s research in the Introduction of the revised paper because we did not want to raise the (false) expectation that we experimentally manipulate intuitive decision making or measure the intuitive nature of the judgments. Instead, we discuss this research in the General Discussion. Specifically, we have added the following passage on page 10, “Moreover, our findings are in line with research on intuitive decision making (e.g., Haidt, 2012), which proposes that individuals have an initial intuitive response towards information provided (e.g., a political statement, or a case of a moral violation). Applied to the present study, it is conceivable that non-supporters of the right-wing populist party have a *negative* intuitive response towards political statements when they are labelled as being endorsed by a right-wing populist party, whereas supporters of right-wing populist parties may have a *positive* intuitive response towards such statements when they are labelled as being endorsed by a right-wing populist party.”**

Social Identity Theorists (Tajfel & Turner, 1979 etc.) have explored this very phenomenon and advanced a compelling argument about the underlying process responsible for this mediating effect. One place where this is explained very clearly is John Turner’s 1991 book ‘Social Influence’, in which he argues on the basis of social identity theorizing, that ‘shared social identity’ mediates the persuasiveness of messages. This insight has since been demonstrated experimentally (Greenaway et al, 2015) and qualitatively in research into populist political messaging (Mols, 2012).

The idea that source information matters was also discussed in quite some detail in a recent paper in Evidence & Policy (Mols, Bell & Head, 2020). More specifically, in this paper it is argued that people’s receptiveness to ‘research evidence’ is mediated by shared social identity. This paper is useful because it considers real life scenarios (e.g. change climate skeptics who identify with a populist party and collectively despise climate researchers will continue to reject evidence of climate change, no matter how much additional research evidence will be presented to them).

**We thank the reviewer for sharing these inspiring ideas. We agree that social identity can play a significant role in the present context. We have thus included a social identity perspective in the General Discussion where we write on page 12, ”The present research can further be discussed from a social identity perspective (e.g., Tajfel & Turner, 1979). Based on social identity theory, it has been argued that a shared social identity of a sender and receiver of a message influences the persuasiveness of the message (e.g., Cohen, 2003; Mols, Bell & Head, 2020). This idea has since received empirical support in experimental (Greenaway et al, 2015) and qualitative (Mols, 2012) research on populist political messaging. Applied to the present study, it is plausible that the logo of the right-wing populist party has activated supporters’ subjective categorization and sense of belonging to the group of right-wing party supporters. Thus, the findings might be traced back to an expression of ingroup favoritism manifested in increased positive evaluation of the political statements when labelled correspondingly. Conversely, the logo of the right-wing populist party may have also activated non-supporters’ subjective categorization and sense of belonging to the group of opponents to right-wing party supporters. As a downstream consequence, these individuals might have engaged in outgroup derogation manifested in decreased positive evaluation of the political statements when labelled as coming from the right-wing populist party. However, these are all post-hoc speculations that need further empirical investigation.”**

Considering the above, it would be good to (a) refrain from claims about ‘being the first to study the effect of labeling on populist messages’, (b) avoid describing the study being reported as about ‘labelling’ and to describe it instead as a study examining whether source information mediates the persuasiveness of a populist message, (c) insert a literature review of studies examining the effect of source information on message persuasiveness (as per above), and (d) to mention ‘shared social identity’ as a likely factor in the section contemplating what could be the ‘underlying process’.

**We are thankful for these useful suggestions, which definitely benefited the paper. As outlined above, we have revised the manuscript accordingly.**

3. In my view, the paper not only requires a proper literature review section (as per above suggestions), but also a section ‘limitations of the present research’. More specifically, it would be good if the authors could reflect on what experiments can tell us about real-life contexts. As Mols & ‘t Hart (2017) explain in their chapter ‘Political Psychology’, experimental research can be extremely useful for examining effects in a controlled environment (to test common assumptions and to debunk common myths). However, all too often researchers project the findings onto real-life phenomena without ever questioning the appropriateness of ‘methodological individualism’ (using individual-level findings to make claims about collective-level phenomena). It would in my view be good to explore such issues in a ‘limitations of the present research’ section, and to at least demonstrate a healthy level of awareness of these rather well-known methodological challenges and pitfalls.

**We thank the reviewer for this useful advice. We now include an explicit limitation section starting on page 11 using the header “Theoretical Implications, Limitations, and Directions for Future Research”**

**We discuss the issue of what experimental basic research can tell us and to what extent it is limited in the General Discussion as follows (p.13): “Notwithstanding the importance of these potential societal implications, a critical note about what experiments—like the one presented in this contribution—can tell us about real-life contexts and phenomena seems in order (see also, e.g., Mols & ‘t Hart, 2017). With the present experimental research, we were able to examine our main hypothesis in a controlled environment, testing whether individuals’ agreement with political statements depends on labelling of the statements’ source. In this regard, we applied an analysis on the level of the individual (reflecting “methodological individualism”, Arrow, 1994). This perspective, however, neglects the dynamic interplay between the individual and the societal/macro level. Therefore, the direct projection of our findings onto real-life phenomena such as the dynamic rise and fall of right-wing populist parties in societies might be limited. That said, it is our hope that the present findings will encourage future research to also study the observed phenomena in a more applied setting while also taking the societal/macro level into account.”**

In sum, in my view this is a promising paper, one that has the potential to make a valuable contribution to the ‘communication’ and ‘messaging’ literatures. However, as it stands the paper suffers from a number of shortcomings. None of these would seem fatal. Rather, in my view the issues I raised could be addressed relatively easily in a revision. I wish the authors good luck with their revision, and I hope my suggestions will prove useful.

**Thank you for these supporting words.**

**Reviewer 2**

The authors investigated whether labelling political statements as endorsed by a right-wing populist party influences people’s agreements with such statements. The research question is comprehensible and straight forward. The empirical part is neat and well done. The results are interesting and communicated in a clear and comprehensible way. I highly recommend publishing this paper.

**We are pleased about the overall positive evaluation.**

My only suggestion is to add a further potential explanation for the labelling effect. Social Identity Theory might predict the same result. The party label might trigger social identification and, related to this, make intergroup conflicts between parties and partisans more salient. Nice paper! Congrats.

**We thank the reviewer for sharing these inspiring ideas relating to social identity. As also mentioned in our responses to Reviewer 1’s comments, we agree that social identity can play a significant role in the present context. We have taken a social identity perspective in the General Discussion where we write on page 12, ”The present research can further be discussed from a social identity perspective (e.g., Tajfel & Turner, 1979). Based on social identity theory, it has been argued that a shared social identity of a sender and receiver of a message influences the persuasiveness of the message (e.g., Cohen, 2003; Mols, Bell & Head, 2020). This idea has since received empirical support in experimental (Greenaway et al, 2015) and qualitative (Mols, 2012) research on populist political messaging. Applied to the present study, it is plausible that the logo of the right-wing populist party has activated supporters’ subjective categorization and sense of belonging to the group of right-wing party supporters. Thus, the findings might be traced back to an expression of ingroup favoritism manifested in increased positive evaluation of the political statements when labelled correspondingly. Conversely, the logo of the right-wing populist party may have also activated non-supporters’ subjective categorization and sense of belonging to the group of opponents to right-wing party supporters. As a downstream consequence, these individuals might have engaged in outgroup derogation manifested in decreased positive evaluation of the political statements when labelled as coming from the right-wing populist party. However, these are all post-hoc speculations that need further empirical investigation.”**
